# Alcohol-impaired driving in US counties, 2002–2012

**DOI:** 10.1186/s12963-018-0158-4

**Published:** 2018-02-01

**Authors:** Jacob E. Sunshine, Laura Dwyer-Lindgren, Alan Chen, Sam R. Sharar, Erin B. Palmisano, Eileen M. Bulger, Ali H. Mokdad

**Affiliations:** 1 Department of Anesthesiology & Pain Medicine, University of Washington School of Medicine, Seattle, USA; 2 Institute for Health Metrics and Evaluation, University of Washington, Seattle, USA; 3 Harborview Injury Prevention and Research Center, University of Washington, Seattle, USA; 4 Department of Surgery, Harborview Medical Center, University of Washington, Seattle, USA

**Keywords:** Alcohol, Motor vehicle crash, Impaired driving, Trauma, Public health

## Abstract

**Background:**

Excessive alcohol consumption and alcohol-impaired driving remain significant public health problems, leading to considerable morbidity and mortality, particularly among younger populations.

**Methods:**

Using data from the Behavioral Risk Factor Surveillance System (BRFSS), we employed a small areas modeling strategy to estimate the county-level annual prevalence of alcohol-impaired driving in every United States county for the years 2002 through 2012, the latest year in which county identifiers were publicly available.

**Results:**

Alcohol-impaired driving episodes declined from 157.0 million in 2002 (prevalence 3.8%: 95% uncertainty interval [UI], 3.7%–4.0%) to 129.7 million in 2012 (prevalence 3.7%: 95% UI, 3.5%–3.8%), a 17.4% decline. There is considerable variation in the prevalence of alcohol-impaired driving at the county level, ranging from 2.0% in the Sitka City Borough of Alaska to 9.3% in Nance County, Nebraska. Clusters of increased alcohol-impaired driving were observed in Northern Wisconsin (Marinette, Florence, Forest, Vilas, Oneida, Iron counties), North Dakota (Cavalier, Pembina, Walsh, Ramsey, Nelson, Benson, Eddy counties) and Montana (Sheridan, Daniels, Roosevelt, Valley, Phillips, Petroleum, Garfield counties).

**Conclusions:**

This study showed guarded progress with respect to the occurrence of alcohol-impaired driving episodes in the US from 2002 to 2012. Because these data rely on self-report, this likely represents an underestimate of the true prevalence of alcohol-impaired driving in the US. As the US continues to have several million episodes of alcohol-impaired driving each month, renewed efforts are needed to mitigate this high-risk health behavior.

## Background

In 2014, motor vehicle crashes (MVCs) remained the leading cause of death for people age 16–24 and among all age groups caused approximately 2 million injuries [1]. In 2015, over 35,000 people lost their life in a MVC in the United States, representing the largest percentage year-over-year increase (7.2%) in fatalities since 1966 [2].

Aside from vehicle speed, the most significant and measureable risk factor for involvement in, and harm from, MVCs is alcohol-impaired driving (AID) [3]. In the US, alcohol impairment is involved in roughly one-third of all MVC fatalities [4] and is an independent risk factor for increased severity of motor collision injuries [5]. Recent estimates for annual costs associated with motor vehicle crashes involving alcohol are approximately $44 billion [6].

Alcohol-impaired driving has been a serious public health problem for decades [4, 7]. Despite previous estimates of over 100 million annual episodes of alcohol-impaired driving nationwide, only approximately 1.1 million arrests were made for driving under the influence (DUI) in 2014 [8, 9]. Although previous reports on alcohol-impaired driving provide national- and state-based estimates, considerable injury prevention and traffic enforcement occur at the municipal level; thus, county-level information is useful to help guide allocation of limited public health resources and to support targeted injury prevention efforts. Using data from the Behavioral Risk Factor Surveillance System (BRFSS), we sought to estimate the county-level annual prevalence of alcohol-impaired driving in every US county for the years 2002 through 2012.

## Methods

### Data source and acquisition

The BRFSS is a state-based, random-digit dialing telephone survey conducted annually by state health departments with support from the Centers for Disease Control and Prevention. The survey collects data on health risks from noninstitutionalized adults aged ≥18 years. Data are collected from a representative sample of the population in each state. The sampling is designed to provide national estimates when each state’s data are combined. Details of BRFSS methodology, including further discussion of sampling and processing, have been described previously [10].

Respondents were asked, “During the past 30 days, how many times have you driven when you’ve had perhaps too much to drink?” We classified respondents into two groups based on their answer: alcohol-impaired drivers as those who answered positively and non-offenders as those who said “none”. Respondents who answered “don’t know/not sure” or “refused” were omitted from the analysis.

Because alcohol-impaired driving is associated with excessive drinking behavior [8], we also calculated the prevalence of binge drinking from the years 2002–2012 at the individual level. At the individual level, binge drinkers were identified in the BRFSS by the following questions: “Considering all types of alcoholic beverages, how many times during the past 30 days did you have 5 or more drinks on an occasion?” (2002–2005) and “Considering all types of alcoholic beverages, how many times during the past 30 days did you have X [X = 5 for men, X = 4 for women] or more drinks on an occasion?” during 2006–2012.

We also used information on respondent age, gender, race/ethnicity (white non-Hispanic, black non-Hispanic, native non-Hispanic, or Hispanic), county of residence, marital status (currently married, never married, or formerly married) and education status (less than high school, high school graduate, some college, or college graduate). Respondents who had missing values for any of these covariates were excluded from the analysis. In 2012, the state-level BRFSS response rate ranged from 24.8% to 59.9% [11].

To ascertain national estimates of alcohol-impaired driving episodes, we calculated the monthly average number of self-reported AID episodes. Self-reported AID episodes are defined as a discrete episode of self-reported episode of alcohol-impaired driving. AID prevalence is defined as the percentage of adult persons in the defined time and county who self-report engaging in alcohol impaired driving at least once in the previous month. Since BRFSS is a monthly telephone survey we then multiplied the monthly average by 12 to obtain the annual number of episodes. Relative risks were calculated using the survey package in R (version 3.1.3) to account for the BRFSS’s complex sample design [12].

### Small area estimation model

We utilized a previously validated small area modeling strategy to estimate the prevalence of reported alcohol-impaired driving in each US county [13]. These models are constructed such that they “borrow strength” across time, space, and from separate data sources (i.e., covariates) in order to maximize the amount of information available for each US county. Briefly, the models were specified as follows:

$$ {Y}_{j,t,a,r,m,e}\sim \mathrm{B}\mathrm{inomial}\left({p}_{j,t,a,r,m,e},{N}_{j,t,a,r,m,e},\right) $$

$$ \mathrm{logit}\left({p}_{j,t,a,r,m,e}\right)={\beta}_0+{\beta}_{1,a}+{\beta}_{2,r}+{\beta}_{3,m}+{\beta}_{4,e}+{\boldsymbol{\beta}}_{\mathbf{5}}\cdotp {\boldsymbol{X}}_{\boldsymbol{j},\boldsymbol{t}}+{u}_j+{w}_t+{d}_{j,t.} $$

where *N*_*j*, *t*, *a*, *r*, *m*, *e*_ indicates the total number of respondents; *Y*_*j*, *t*, *a*, *r*, *m*, *e*_ indicates the number of individuals who report alcohol-impaired driving; and *p*_*j*, *t*, *a*, *r*, *m*, *e*_indicates the true prevalence of alcohol-impaired driving in county *j,* year *t,* age group *a,* race/ethnicity group *r*, marital status group *m*, and education group *e.*

The *β* terms represent fixed effects: *β*_0_ is the intercept; *β*_1, *a*_ represents age-group effects, which account for differences in alcohol-impaired driving by age; *β*_2, *r*_, *β*_3, *m*_, and *β*_4, *e*_ represent effects of race/ethnicity, marital status, and education, respectively, and are utilized to account for differences in reported alcohol-impaired driving among each of these different groups. *β*_5_ is a vector of coefficients on six county-level covariates that are thought to be predictive of alcohol-impaired driving (alcohol stores and places per capita, percent of the population living below the poverty line, the unemployment rate, county population density, county education level, and racial composition).

The final terms represent random effects: *u*_*j*_ and *w*_*t*_ are county- and year-level random effects, respectively; *d*_*j*, *t*_ is a county-year random effect. All random effects terms are presumed to follow a conditional autoregressive distribution [14, 15]. Of note, the spatial, temporal, and spatial-temporal random effects are all assigned the same conditional autoregressive distribution [14]; the only difference is that “neighbors” are defined in terms of queen contiguity for the spatial effects, and by adjacent years for temporal effects.

The Template Model Builder package from R version 3.2.4 was used to construct the models [16, 17]. We simulated 1000 draws of *p*_*j*, *t*, *a*, *r*, *m*, *e*_ from the posterior distribution. The draws were post-stratified by education, race, and marital status utilizing population counts from the US Census and American Community Survey. This was done to ensure that prevalence estimates represent the demographic composition of a county, even when a county’s demographic groups may have limited or no survey responses.

Draws were age-standardized using the 2010 census population. Posterior point estimates were derived from the mean of the 1000 draws; 95% uncertainty intervals (UI) were derived using the 2.5th and 97.5th percentiles. State and national estimates were calculated by population-weighting the county-level estimates.

Data were de-identified and publicly available, therefore institutional review board approval was not required. All analyses were completed in 2016.

## Results

From 2002 to 2012, binge drinking episodes increased nationally from an estimated 1,703,955,652 annual episodes to 1,927,818,363 annual episodes, respectively, an 13.1% change. Respondents who reported binge-drinking behavior were more likely to engage in alcohol-impaired driving compared to drinkers who do not binge (relative risk 11.0, 95% confidence interval [CI], 9.9–12.2).

During the years in which the BRFSS asked respondents about alcohol-impaired driving (2002, 2004, 2006, 2008, 2010, 2012) there were an estimated 851,902,397 episodes of reported alcohol-impaired driving in the US. Using the annual mean from these biennial estimates, that equates to approximately 1,561,821,061 total reported episodes of alcohol-impaired driving during the study period, 2002–2012.

**Fig. 1:**
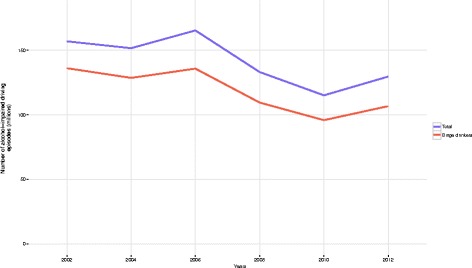
Reported episodes (in millions) of alcohol-impaired driving overall, and by those who also reported binge drinking: United States, 2002–2012

In 2002, there were an estimated 156,963,735 reported episodes of alcohol-impaired driving; in 2012, there were an estimated 129,652,146 reported episodes of alcohol-impaired driving, a change of − 17.4% (Fig. 1). In 2002, the age-standardized prevalence of alcohol-impaired driving in the US was 3.8% (95% UI, 3.7–4.0), compared to 3.7% (95% UI, 3.5%–3.8%) in 2012. Men were more likely to engage in alcohol-impaired driving than women; in 2012, men had 102.6 million episodes of alcohol-impaired driving compared to 27.1 million episodes among women (879 episodes per 1000 adult males versus 219 episodes per 1000 adult females).

Impaired driving among under age (< 21 years old) adults has decreased substantially since 2002. Among males, in 2002, the 18–20 year old population accounted for 14,848,467 reported episodes (2447 episodes/1000 population) of alcohol-impaired driving compared to 5,836,464 reported episodes (764 episodes/1000 population) in 2012, a 60.7% decline; among females, in 2002, the 18–20 year old population accounted for 2,656,167 reported episodes (503 episodes/1000 population) of alcohol-impaired driving compared to 1,378,562 (195 episodes/1000 population) in 2012, a 48.1% decline.

**Fig. 2:**
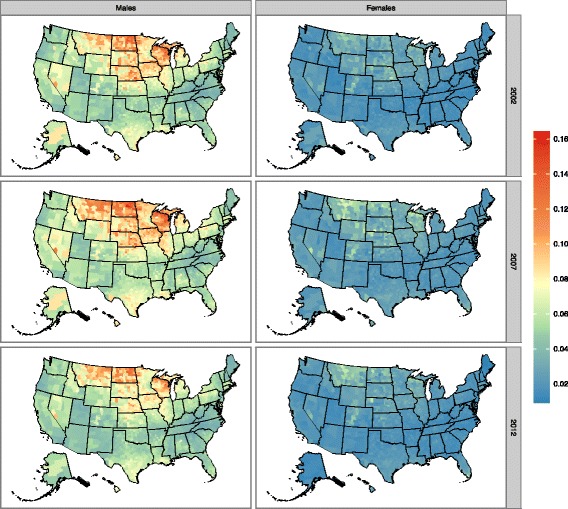
Age-standardized prevalence of reported alcohol-impaired driving by US county, 2002, 2007, and 2012

There is considerable variation in the prevalence of alcohol-impaired driving at the county level (Fig. 2). In 2012, among US counties, the prevalence of alcohol-impaired driving ranged from 2.0% in the Sitka City Borough of Alaska to 9.3% in Nance County, Nebraska. Clusters of increased alcohol-impaired driving were observed in Northern Wisconsin (Marinette, Florence, Forest, Vilas, Oneida, Iron counties), North Dakota (Cavalier, Pembina, Walsh, Ramsey, Nelson, Benson, Eddy counties) and Montana (Sheridan, Daniels, Roosevelt, Valley, Phillips, Petroleum,Garfield counties) (Fig. 2).

**Fig. 3:**
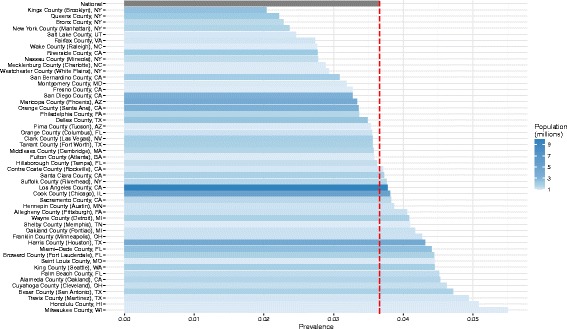
Age-standardized prevalence of reported alcohol-impaired driving: 50 most populous US counties, 2012. County seat noted in parentheses when different from county name

We also examined the prevalence of alcohol-impaired driving in the 50 most populous US counties (Fig. 3).

Among the 50 most populous US counties, Kings County, New York, had the lowest prevalence of alcohol-impaired driving (prevalence 2.0%, 95% UI, 1.5%–2.7%). The lowest prevalence counties that were not within boroughs of New York City were Salt Lake County, Utah (prevalence 2.4%, 95% UI 2.0%–2.9%) and Fairfax County, Virginia (prevalence 2.7%, 95% UI 2.1%–3.4%). Among the 50 most populous US counties, the county with the highest prevalence of alcohol-impaired drivers was Milwaukee County, Wisconsin (prevalence 5.5%, 95% UI, 4.6%–6.6%), followed by Honolulu County, Hawaii (prevalence 5.1%, 95% UI, 4.1%–6.3%).

## Discussion

This study found guarded progress with respect to the number of episodes of reported alcohol-impaired driving in the US from 2002–2012. However, while the age-standardized prevalence of this behavior has decreased slightly, there continues to be several million episodes of alcohol-impaired driving each month. Our findings call for renewed efforts to decrease episodes of alcohol-impaired driving in order to reduce mortality and morbidity from motor vehicle crashes.

Our results likely represent the tip of the iceberg with respect to the true prevalence of alcohol-impaired driving, as the data presented here rely on self-reported behavior, which in this circumstance is subject to social desirability bias and legal issues. Indeed, because people may not report a criminal behavior during a telephone survey, what is reported here likely represents a significant underestimate of the true prevalence of alcohol-impaired driving.

Excessive consumption of alcohol has several adverse effects on health, including increased risk for cardiovascular diseases, cancers, violence, traumatic injury, liver disease and more [18–25]. Our study revealed persistently high levels of alcohol-impaired driving in the US. This coupled with the health danger of heavy drinking underscores the need for refocused efforts to reduce excessive drinking and alcohol-impaired driving. Indeed, the US would greatly benefit from a renewed, comprehensive approach to mitigate excessive alcohol use and impaired driving. Such an approach would need to involve improved screening, prevention, and treatment from health care workers, in addition to enhanced efforts from public health officials, city, state, and federal governments, law enforcement and, commercial enterprises focused on transportation.

Physicians can play a role in mitigating this high-risk health behavior. The United States Preventive Services Task Force recommends screening patients for risky drinking behaviors and, for those screening positive, to provide evidence-based, brief behavioral counseling interventions [26]. Such counseling may indirectly decrease the risk of alcohol-impaired driving; indeed, over 80% of reported alcohol-impaired driving episodes were reported by people who engage in binge drinking [8, 27]. Physicians can also help protect those put at risk by alcohol-impaired drivers, for example by counseling patients about the importance of safety restraint devices, such as car seats for children and 100% compliance with seatbelt utilization [28].

Physicians can also play a role in reducing subsequent harmful behavior from alcohol in the hospital setting, following an alcohol-related traumatic injury. The American College of Surgeons, which develop mandates for US trauma centers, requires that Level I trauma centers have adequate resources to both identify problem drinkers and provide behavioral interventions when possible [29]. These interventions, and the training underlying them, have been found to be both beneficial to patients and to providers [30, 31].

Despite the scope of this public health problem, there are reasons to believe improvements can be made through focused efforts by city, state, and federal governments. Indeed, in light of recent spikes in motor vehicle fatalities, there is renewed focus on preventive measures to reduce and eliminate motor vehicle crash fatalities. The clearest manifestation of these recent efforts in the US is the launch of *Road to Zero,* a multi-agency federal initiative whose goal is to minimize mortality from motor vehicle crashes and eliminate it completely by 2046 [32].

Other advanced countries, including those with large geographic landmass, have shown the ability to reduce mortality from motor vehicle crashes, including from alcohol-impaired driving [3]. Several countries employ best-practices, for instance alcohol-impaired driving laws based on blood alcohol content (BAC) limits less than or equal to 0.05 g/dl [33]; in the US, transportation-related federal funding remains tied to BAC limits of 0.08 g/dl [34].

Other governmental best practices may specifically target younger, novice drivers, who have an increased risk of crashing when impaired by alcohol compared to more experienced drivers [35]. These measures include graduated licensing laws, whereby novice drivers acquire increasing driving privileges over time, as well as a reduction of the BAC limit for novice drivers to 0.02 g/dl or zero [36]. Both of these interventions have been shown to reduce the risk of crash and death from alcohol-impaired driving, yet each has limited adoption nationwide. Other potential ways for local jurisdictions to reduce excessive alcohol consumption is by reducing the density of alcohol outlets at the neighborhood level [37, 38].

Technologies that enable transportation alternatives (i.e., ridesharing platforms such as *Uber Technologies* and *Lyft*) theoretically hold promise for reducing fatalities from alcohol-impaired driving, however the most comprehensive study to date found no significant effect on fatality rates in markets where these services exist [39]. As these services expand and mature, it remains to be seen if they have the ability to significantly reduce the number of alcohol-impaired driving episodes or impact fatality rates.

There are several limitations to this study. First, BRFSS data are based on self-reports, and in our case we may have underestimated the true rates of alcohol consumption and driving while impaired. Second, the BRFSS is a cross sectional study and therefore we are unable to determine causation. Third, BRFSS response rates (similar to most public surveys) have been falling, leaving open the possibility that non-response could affect our results. The BRFSS has, however, been shown to provide reliable and valid estimates in the context of declining response rates [40]. Moreover, the BRFSS is based on a large sample size and a standard methodology that allows comparability across geographic areas and time.

## Conclusion

From 2002 to 2012, guarded progress occurred with respect to the annual number of reported alcohol-impaired driving episodes in the US. However, there continues to be several million episodes of alcohol-impaired driving each month, with notable clusters of high-risk counties located in the Middle West and Great Plains regions of the US. Because these data rely on self-reporting, this likely represents a significant underestimate of the true prevalence of alcohol-impaired driving in the United States. As levels of alcohol-impaired driving remain persistently high, renewed focus is needed to mitigate this high-risk health behavior.
